# Assessment of patients’ knowledge and perceptions of MRI scans and safety in Saudi Arabia

**DOI:** 10.3389/fpubh.2024.1439131

**Published:** 2024-08-05

**Authors:** Sami A. Alghamdi

**Affiliations:** Radiological Sciences Department, College of Applied Medical Sciences, King Saud University, Riyadh, Saudi Arabia

**Keywords:** knowledge, MRI, safety, patient, Saudi Arabia

## Abstract

**Background:**

This study was conducted to assess the levels of knowledge about MRI scans and safety measures among patients in Saudi Arabia.

**Methods:**

This cross-sectional study was conducted at nine Saudi Arabian hospitals and utilized a questionnaire comprising 22 items that evaluated patients’ knowledge regarding MRI scans and safety measures, divided into four sections. The questions encompassed patients’ sociodemographic data (A), knowledge about MRI (B), safety measures (C), and communication (D). Descriptive statistics were used to characterize the participant demographics and responses.

**Results:**

Out of 446 MRI patients, 60.5% correctly identified that MRI does not involve ionizing radiation, and 78% recognized MRI as a diagnostic tool. Further, 94.2% knew that metal objects are not allowed in MRI rooms. However, 80.3% incorrectly believed that pregnant patients cannot undergo MRI at any time, 57% thought the MRI scanner is turned off when not in use, and 72.6% did not recognize any MRI-compatible devices. About 62% were unaware of the need for kidney function tests with contrast agents, and 43% reported anxiety during MRI scans. Overall, 57% of the patients had limited knowledge of MRI safety, with 39.5% considering their understanding adequate. Educational attainment and employment status were significantly associated with improved MRI knowledge. Most participants sought information from healthcare professionals.

**Conclusion:**

This study highlights the need to educate patients about MRI procedures and safety protocols. Significant gaps remain in patients’ knowledge, especially regarding safety measures. Higher levels of educational attainment and employment status were linked to greater levels of MRI knowledge, suggesting the importance of targeted educational interventions. Healthcare professionals were the patients’ main information sources; nevertheless, comprehensive and accessible information is necessary. Improved communication and training for healthcare providers can enhance patient understanding and experiences during MRI scans.

## 1 Introduction

Magnetic resonance imaging (MRI) is a high-resolution cross-sectional imaging modality that provides precise, detailed anatomical images of the human body (1). It is non-invasive and does not alter the structure, composition, or physical properties of atoms, unlike other imaging techniques involving ionizing radiation modalities (2). However, clinical MRI is associated with several potential hazards, including an increased risk of damage due to the projectile effects of strong static magnetic fields. Furthermore, humans are impacted by time-varying gradient fields, which manifest in the form of acoustic noise and can cause unintended peripheral nerve stimulation (3, 4). Tissue heating and concerns over the specific absorption rate (SAR), which is a measure of RF energy absorption that can lead to high temperatures, are other hazards associated with radiofrequency (RF) fields. In addition, the increasing utilization of various medical devices and implants in the human body has made MRI safety more complex than before, as there are increased risks associated with implants and foreign objects, which can interact with the magnetic fields and potentially cause harm or malfunction (5). Ensuring MRI safety is of utmost importance in protecting patients, MRI staff and other medical personnel, and equipment (3, 6).

Assessing patients’ perceptions and knowledge of MRI scans and safety measures is crucial for ensuring the successful implementation of this diagnostic imaging modality. Patients’ awareness and understanding of the MRI procedure and its safety aspects contribute significantly to their cooperation, reducing the likelihood of complications and enhancing the overall effectiveness of the process. Several studies that have investigated patients’ knowledge levels and perceptions regarding MRI scans have highlighted the need for targeted educational interventions (7–9).

Understanding patients’ anxiety levels and concerns related to MRI is a key aspect of assessing their perceptions of this procedure. Patients may experience anxiety due to the enclosed nature of the MRI machine, the loud noises produced during the scan, or fear of the unknown. Research has shown that addressing these concerns through pre-scan education and communication significantly improves patient comfort and cooperation during MRI procedures (2, 10).

In addition, evaluating patients’ knowledge of MRI safety is essential to prevent adverse events. Safety concerns often arise from misconceptions about the magnetic field, potential interactions with metallic objects, and the use of contrast agents (8, 11). Assessing patients’ understanding of safety protocols and providing clear information on contraindications can contribute to minimizing the risks associated with MRI.

Healthcare providers play a pivotal role in enhancing patients’ awareness and knowledge of MRI. Effective communication strategies, including the use of informational pamphlets, videos, and personalized discussions, have been shown to positively impact patients’ perceptions and understanding of the MRI procedure (12). By incorporating patient education into routine clinical practice, addressing concerns, and facilitating effective communication, healthcare professionals can ensure that patients are informed and cooperative during MRI scans (2, 13).

Understanding patients’ knowledge and perceptions is crucial for safe and effective MRI procedures. This study aims to identify specific gaps in patient knowledge and misconceptions about MRI, which can be addressed through targeted educational interventions and improved communication strategies by healthcare providers. By highlighting these gaps, the study contributes to the development of more effective patient education programs and safety protocols. Accordingly, the purpose of this study was to assess patients’ knowledge and perceptions of MRI scans and safety in Saudi Arabia. By incorporating the findings into clinical practice, healthcare providers can optimize patient care and promote positive and safe MRI environments.

## 2 Methods

### 2.1 Participants and recruitment

This cross-sectional study, conducted via a questionnaire, took place in January and February 2024 at nine hospitals in Saudi Arabia, including two university hospitals, one National Guard hospital, three public hospitals under the Saudi Health Ministry, and three private hospitals. The hospitals were selected to represent the main health sectors of the nation and were located in six different provinces: Riyadh, Makkah, the Western Region, Asir, Hail, and Albaha. The geographic and demographic diversity of the hospitals allowed for capturing the experiences of the patient population across various healthcare settings in the country.

To enhance the data collection process, four undergraduate radiological students and seven radiological technologists were trained to conduct interviews with MRI outpatients. This training included standardized data collection procedures, ethical guidelines, and techniques to avoid bias. They were also instructed on clearly explaining each question to respondents to ensure accurate understanding, ensuring consistency and reliability in the data collection process.

Following their MRI procedures, patients in the MRI departments of the selected hospitals were given a quick response code (QR code) to access the survey on their electronic devices. To cater to different preferences, the survey was available both online and in paper form. Participation was voluntary, and participants were briefed on the study’s purpose and assured of their confidentiality. Consent was garnered from each participant via a form included in the survey questionnaire.

### 2.2 Survey

The questionnaire was adapted from two previous studies (8, 11) and presented in Arabic. It comprised 22 items divided into four sections that aimed to evaluate patients’ knowledge of MRI scans and safety measures, encompassing patients’ sociodemographic data (A) and knowledge about MRI (B), safety measures (C), and communication (D). The reliability and effectiveness of the questionnaire in assessing patients’ knowledge in this domain were validated. The reliability was assessed by calculating the Cronbach’s alpha value, which was 0.582 (*p* < 0.001), indicating a moderate level of statistically significant internal consistency. The validity was evaluated using the Kaiser-Meyer-Olkin (KMO) measure, with a value of 0.806 (*p* < 0.001), suggesting substantial correlation within the data.

In this cross-sectional study, data was collected from nine hospitals in Saudi Arabia over a two-month period. The participating hospitals reported a daily outpatient turnover of 20–40 patients on average for MRI scans. To ensure statistical robustness and precision, the required sample size was calculated based on a 95% confidence level and a 5% margin of error and determined to be approximately 385 MRI patients. This sample size was deemed sufficient to provide reliable and generalizable results and to represent a significant and manageable subset of the overall patient population during the study period.

### 2.3 Ethical approval

Ethical guidelines were stringently adhered to throughout the study. The necessary ethical clearance was obtained from the institutional review board at King Saud University, Medical City (Reference No. E-24-8424), and informed consent was secured from all participants prior to their involvement in the study.

### 2.4 Statistical analysis

Data were analyzed using IBM SPSS software for Windows version 26.0 (IBM Corp., Armonk, N.Y., USA). Descriptive statistics (frequencies and percentages) were used to describe the categorical variables. Pearson’s chi-square test was used to test for homogeneity and associations, and odds ratios were used to measure the associations between the categorical sociodemographic variables and level of MRI knowledge. A binary multivariate logistic regression was used to identify the independent factors associated with the level of MRI knowledge. *p*-values ≤0.05 and 95% confidence intervals were used to report the statistical significance and precision of the results.

## 3 Results

A total of 446 participants were involved in this study after 96 conveyed their refusal. Among them, 35.4% were 26–40 years of age, and 30.5% were 41–55 years. Male participants constituted 53.8% of the sample, and over half of the participants were married. A high proportion (47.5%) had a bachelor’s degree, and 47.1% were employed (Table 1).

**Table 1:** Sociodemographic characteristics of the study participants (*n* = 446).

Characteristics	No. (%)
Age group	
18–25	96 (21.5)
26–40	158 (35.4)
41–55	136 (30.5)
≥ 56	56 (12.6)
Gender	
Male	240 (53.8)
Female	206 (46.2)
Marital status	
Unmarried	130 (29.1)
Married	260 (58.3)
Divorced	40 (9.0)
Widowed	16 (3.6)
Educational status	
Primary, intermediate, and secondary school	104 (23.3)
Diploma	56 (12.6)
Bachelor’s degree	212 (47.5)
Master’s degree	48 (10.8)
PhD degree	26 (5.8)
Employment status	
Employed	210 (47.1)
Retired	36 (8.1)
Freelance	24 (5.4)
Unemployed	46 (10.3)
Housewife	80 (17.9)
Student	50 (11.2)

A comparison of participants’ responses to items related to knowledge about MRI can be observed in Table 2. Notably, 64.6% of the participants had previously undergone an MRI scan—a statistically significantly higher proportion than those who had not (*p* < 0.001). For the item, “Does MRI use ionizing radiation like a CT scan,” 60.5% of the respondents provided the correct answer, “no,” which was statistically significantly greater than any other response (*p* < 0.001), indicating that a high proportion of participants possessed appropriate knowledge. Regarding the item, “Will MRI diagnose or treat your disease,” 78% of the study subjects correctly responded “diagnose,” which was statistically significantly higher than the other response (*p* < 0.001). Regarding the item, “How would you rate your understanding of the purpose of the MRI scan,” only 26.4% of the responses were “good” or “excellent,” whereas 33.6% were “poor,” and 33.6% were “average”; the differences in the responses for this item were statistically significant (*p* < 0.001) (Table 2).

**Table 2:** Comparison of participants’ responses regarding their knowledge about MRI.

Items of knowledge	No. (%)	Χ^2^-value	*p*-value
Have you done an MRI scan before?			
Yes	288 (64.6)	37.89	< 0.001
No	158 (35.4)		
Does MRI use ionizing radiation like a CT scan?			
Yes	176 (39.5)	19.81	< 0.001
No	270 (60.5)		
Will MRI diagnose or treat your disease?			
Diagnose	348 (78.0)		
Treat	8 (1.8)	423.52	< 0.001
Both	90 (20.2)		
How would you rate your understanding of the purpose of the MRI scan?			
Very poor	28 (6.3)		
Poor	150 (33.6)		
Average	150 (33.6)	151.8	< 0.001
Good	76 (17.0)		
Excellent	42 (9.4)		

An analysis and comparison of the participants’ knowledge of MRI safety measures (see Table 3) revealed statistically significant variations in the response rates for eight items. Regarding the question, “Can pregnant patients undergo MRI scans at any time,” 80.3% incorrectly answered “no.” For the query, “Do you think that the MRI scanner remains on when there are no patients, “57% incorrectly said “no.” A large proportion, 72.6%, did not recognize the distinction between MRI-compatible and MRI-incompatible services. However, a notably high percentage of subjects (94.2%) correctly responded “no” to the safety question “Do you think you are allowed to take metal items into the MRI scan room?.” About 62.3% were unaware of why patients requiring MRI with contrast agents must undergo kidney function tests. Further, 81.6% were unfamiliar with the four MRI zones. Regarding MRI scan safety measures, approximately 57% gave positive responses. While 39.5% reported their understanding of MRI safety as “enough” or “more than enough,” 52% described it as “very limited” or “limited.” The proportion of responses to the safety measure items were significantly higher than the proportions of other answers within each category. For the item “What kind of problem did you face during the scan period,” most participants reported “anxiety” (43%), followed by “nothing” (33.9%), “headache” (19.3%), and “claustrophobia” (18.8%).

**Table 3:** Comparison of participants’ responses regarding MRI safety measures.

Items of safety	No. (%)	Χ^2^-value	*p*-value
Can pregnant patients undergo MRI scans at any time?			
Yes	88 (19.7)	163.45	< 0.001
No	358 (80.3)		
Do you think that the MRI scanner remains on when there are no patients?			
Yes	192 (43.0)	8.62	0.003
No	254 (57.0)		
Do you know the difference between MRI-compatible and MRI-incompatible services?
Yes	122 (27.4)		
No	324 (72.6)	91.49	< 0.001
Do you think you are allowed to take metal items into the MRI scan room?			
Yes	26 (5.8)		
No	420 (94.2)	348.06	< 0.001
Do you know why patients requiring MRI with contrast agents should undergo a kidney function test?
Yes	168 (37.7)		
No	278 (62.3)	27.13	< 0.001
Are you aware of the 4 MRI zones?			
Yes	82 (18.4)		
No	264 (81.6)	178.3	< 0.001
What kind of problem did you face during the scan period? *			
Anxiety	192 (43.0)		
Headache	86 (19.3)	–	–
Relaxed	88 (19.7)		
Claustrophobia	84 (18.8)		
Nothing	151 (33.9)		
Are you aware of the safety measures associated with MRI scans?			
Yes	254 (57.0)		
No	192 (43.0)	8.62	0.003
How would you describe your knowledge about MRI safety?			
Very limited	38 (8.5)		
Limited	194 (43.5)	235.6	< 0.001
Average	38 (8.5)		
Enough	136 (30.5)		
More than enough	40 (9.0)		

*Multiple responses.

**Figure 1:**
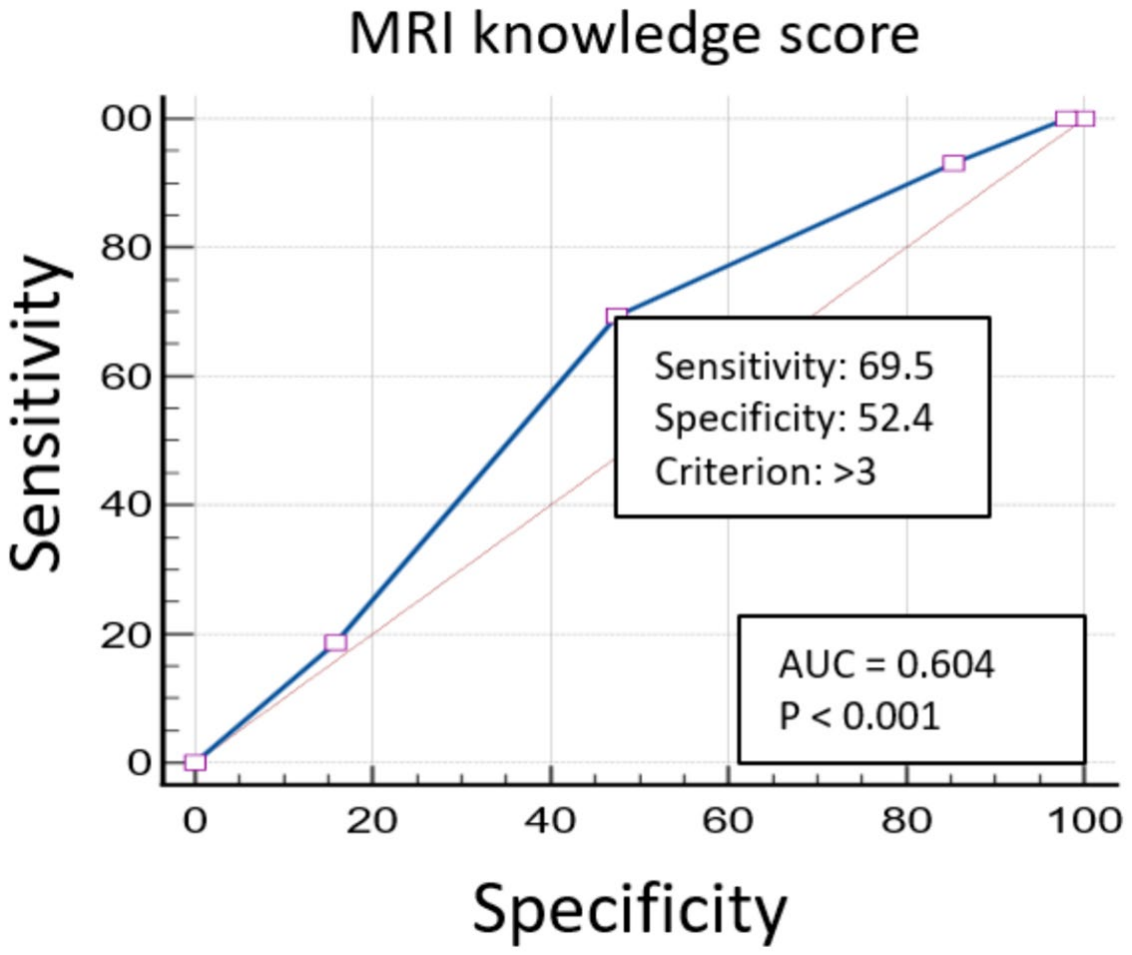
The ROC curve illustrates the performance of the MRI knowledge score. The area under the curve (AUC) is 0.604, with a *p* value of less than 0.001, indicating statistical significance. The sensitivity and specificity for the cutoff criterion of greater than 3 are 69.5 and 52.4%, respectively.

Participants were categorized as having either good or poor knowledge based on their MRI knowledge scores. Specifically, participants with scores greater than 3 (out of 5) were classified as having good knowledge, while those with scores of 3 or less were classified as having poor knowledge. This cutoff point was determined using area under the curve (AUC) analysis. The AUC analysis considered participants’ responses to the question, “How would you rate your understanding of the purpose of the MRI scan?.” These responses were provided on a 5-point Likert scale (very poor, poor, average, good, and excellent). Responses were subsequently dichotomized into two categories: poor and good knowledge. These dichotomized responses served as the gold standard against which the total correct responses to five MRI knowledge questions were evaluated. Figure 1 presents the ROC curve, demonstrating the sensitivity and specificity of the cutoff value.

Further, the MRI knowledge levels were assessed based on their associations with the sociodemographic characteristics of the study subjects. Table 4 shows how educational status and employment status were associated with MRI knowledge levels. Specifically, participants holding a diploma, bachelor’s degree, master’s degree, or PhD had higher odds of having good MRI knowledge than those with primary, intermediate, and secondary school level of education (*p* < 0.001). Further, participants who were employed had higher odds of having good MRI knowledge than those who were unemployed (*p* = 0.009). The other characteristics (age, gender, and marital status) were not significantly associated with MRI knowledge level. The unadjusted and adjusted odds ratios of the significant and nonsignificant variables are given in Table 4.

**Table 4:** Variables associated with MRI knowledge level according to the binary logistic regression analysis.

Variable	MRI knowledge level		Χ^2^-value	*p*-value	Unadjusted odds ratio (95% CI)	Adjusted odds ratio (95% CI)
	Good	Poor				
Age group						
18–25	50 (52.1)	46 (47.9)	7.15	0.067	1.68 (0.86,3.28)	2.87 (0.80, 10.24)
26–40	84 (53.2)	74 (46.8)			1.75 (0.94,3.26)	1.69 (0.59, 4.84)
41–55	82 (60.3)	54 (39.7)			2.35 (1.24,4.44)	2.66 (0.95, 7.45)
≥ 56	22 (39.3)	34 (60.7)			1.0 (ref.)	1.0 (ref)
Gender						
Male	132 (55.0)	108 (45.0)	0.559	0.455	1.15 (0.79,1.67)	0.92 (0.56, 1.51)
Female	106 (51.5)	100 (48.5)			1.0 (ref.)	1.0 (ref)
Marital status						
Unmarried	68 (52.3)	62 (47.7)	3.861	0.277	1.83 (0.63,5.32)	1.07 (0.25, 4.66)
Married	138 (53.1)	122 (46.9)			1.88 (0.67,5.34)	0.93 (0.26, 3.34)
Divorced	26 (65.0)	14 (35.0)			3.10 (0.93,10.30)	1.67 (0.37, 7.50)
Widowed	6 (37.5)	10 (62.5)			1.0 (ref.)	1.0 (ref.)
Educational status						
Primary, intermediate, and secondary school	30 (28.8)	74 (71.2)	48.395	< 0.001	1.0 (ref.)	1.0 (ref.)
Diploma	26 (46.4)	30 (53.6)			2.14 (1.10,4.20)	1.66 (0.80,3.42)
Bachelor’s degree	124 (58.5)	88 (41.5)			3.48 (2.10,5.76)	3.18 (1.81,5.59)
Master’s degree	40 (83.3)	8 (16.7)			12.33 (5.17,29.43)	11.40 (4.41, 29.50)
PhD degree	18 (69.2)	8 (30.8)			5.55 (2.18,14.13)	5.36 (1.92, 14.96)
Employment status						
Employed	128 (61.0)	82 (39.0)	15.38	0.009	2.92 (1.50,5.70)	1.83 (0.84, 4.00)
Retired	20 (55.6)	16 (44.4)			2.34 (0.96,5.73)	4.57 (1.24, 16.84)
Freelance	14 (58.3)	10 (41.7)			2.62 (0.95,7.23)	1.35 (0.43, 4.29)
Student	26 (52.0)	24 (48.0)			2.03 (0.89,4.62)	1.47 (0.56, 3.84)
Housewife	34 (42.5)	46 (57.5)			1.38 (0.65,2.94)	1.94 (0.79, 4.76)
Unemployed	16 (34.8)	30 (65.2)			1.0 (ref.)	1.0 (ref.)

The distribution of participants’ responses regarding communication with healthcare providers about MRI scans can be observed in Table 5. For the item, “Where do you typically seek information about MRI scans and safety,” most participants (86.3%) stated that they obtained information from healthcare professionals; this was followed by the internet (45.7%), friends or family (23.5%) and printed materials (10.3%). For the item, “How satisfied are you with the information provided by healthcare providers before undergoing an MRI scan,” only 15.2% responded that they were “very satisfied.” Further, 80.7% of the participants conveyed that they would like to be given more information about MRI before undergoing the procedure (Table 5).

**Table 5:** Distribution of participants’ responses regarding communication with healthcare providers about MRI scans.

Communication items	No. (%)
Where do you typically seek information about MRI scans and safety?*	
Healthcare professionals	385 (86.3)
Internet	204 (45.7)
Friends or family	105 (23.5)
Printed materials (brochures & pamphlets)	46 (10.3)
How satisfied are you with the information provided by healthcare providers before undergoing an MRI scan?	
Very dissatisfied	9 (2.01)
Dissatisfied	6 (1.3)
Neutral	168 (37.7)
Satisfied	195 (43.7)
Very satisfied	68 (15.2)
Would you like more information about MRI scans before undergoing the procedure?	
Yes	360 (80.7)
No	86 (19.3)

*Multiple responses.

## 4 Discussion

Magnetic resonance (MR) is a highly valuable and non-invasive diagnostic tool that is widely used in medical imaging. Advancements in MRI technology have led to stronger magnetic fields and higher radiofrequency power, necessitating the establishment of strict safety protocols to protect patients. Educating patients about the MRI process and safety measures is crucial for ensuring their safety, reducing anxiety, and ensuring cooperation (14).

This study involved 446 participants, with the majority aged between 26 and 55 years. Male participants constituted 53.8% of the sample, and over half were married. A significant proportion (47.5%) held a bachelor’s degree, and 47.1% were employed.

The analysis of the participants’ MRI knowledge led to several key findings. Notably, 64.6% of the participants had previously undergone an MRI scan. Regarding their understanding of the purpose of MRI, 60% of the participants rated their understanding as “good,” “average,” or “excellent,” while 40% rated it as “poor” or “very poor.” This indicates that a high proportion of the participants had an optimal, satisfactory level of understanding of the purpose of an MRI scan. When asked whether MRI uses ionizing radiation like a CT scan, 60.5% correctly answered “no,” indicating a good level of knowledge among the subjects; this finding was statistically significant. However, this contrasts with the results of a study conducted by Luca et al. in Italy (15), wherein only 43.0% of respondents correctly classified MRI as a ionizing-free examination. Additionally, 78% of the participants in the present study correctly identified MRI as a diagnostic tool rather than a treatment modality. This contrasts with the findings of a study conducted by Susmita et al. in Nepal (8), wherein 55.2% correctly recognized the function of MRI in disease diagnosis. The higher percentages obtained in our study indicate the relatively high knowledge levels of the participants in our sample compared to the Italian and Nepalese cohorts. This difference may be attributed to variations in educational initiatives, public awareness campaigns, and exposure to MRI information.

The analysis of participants’ knowledge of MRI scan safety measures revealed significant variations in response accuracy across all eight items. Specifically, 80.3% incorrectly reported that pregnant patients cannot undergo MRI at any time, and 57% wrongly stated that MRI scanners remain off when not in use. Additionally, 72.6% did not understand the difference between MRI-compatible and MRI-incompatible devices. However, 94.2% correctly knew that no metal objects are allowed inside the MRI scan room. A large percentage of patients (62.3%) were unaware of the need for kidney function tests before using contrast agents, and 81.6% were unfamiliar with the four MRI zones. When describing their own knowledge of MRI safety, about 57% described their knowledge as “very limited” or “limited.” A study conducted in the Asir region, which involved one hospital in southern Saudi Arabia (16), revealed similar findings, with the participants having poor knowledge about MRI safety. Our study, which was broader and included nine hospitals across Saudi Arabia, also revealed a lack of awareness about MRI safety protocols among the participants. Therefore, targeted educational initiatives and training programs that address the aforementioned lack of knowledge, thereby improving overall awareness and ensuring patient safety, are needed (17).

Anxiety may be triggered by several factors related to the MRI procedure itself, such as the loud noise, the confined space, and lengthy examination times, as well as patient-related concerns, such as fear of pain, potential diagnostic outcomes, and loss of control. Anxiety can lead to increased respiratory rates, bowel movements, and fluid flow, compromising image quality due to movement. This results in the MRI procedure having to be repeated, thereby extending the examination duration and consuming costly resources (18, 19). A high prevalence of anxiety during MRI scans (43%) was reported by the participants in the present study. Grey et al. (20) suggested that anxiety can be reduced through simple measures, such as providing accurate information about MRI machines and procedures, listening to recorded MRI sounds, and visiting the control room beforehand. It is recommended that healthcare professionals prepare and assess patients prior to their MRI scans.

Our comparison of MRI knowledge scores, which were categorized as “good” and “poor” based on the cutoff score of 3, revealed that educational status and employment status were significantly associated with MRI knowledge levels. Participants with higher levels of educational attainment had significantly higher odds of possessing good MRI knowledge. This aligned with the findings of Hossen et al.’s study (21), in which a statistically significant association was found between patients’ knowledge and educational status, with the latter affecting patients’ knowledge of MRI safety. These findings suggest that it would be beneficial for educational interventions on MRI safety to focus on populations with low levels of educational attainment. Similarly, employed subjects were more likely to have good MRI knowledge than unemployed subjects.

The majority of participants (86.3%) sought information about MRI from healthcare professionals, while 45.7% turned to the internet, 23.5% consulted friends or family, and 10.3% relied on printed materials. Approximately 58% reported being “very satisfied” or “satisfied” with the information provided by healthcare providers before undergoing MRI scans, and 80.7% expressed a desire for more information. Thus, although healthcare professionals were the primary sources of information for most patients, a considerable number of participants still felt inadequately informed, highlighting the need for comprehensive information to be provided. Staff training for radiologists, radiology technologists, and nurses in MRI settings would be beneficial in enhancing interpersonal and communication skills as well as their ability to facilitate patient comfort and relaxation. This can help mitigate the potentially stressful or anxiety-provoking aspects of the MRI process, ultimately leading to improved patient experiences and outcomes (22).

This study provides several contributions to patient education and safety measures within the MRI environment. Significant gaps in patients’ knowledge about MRI procedures and safety measures were identified, highlighting areas where patient education needs to be strengthened. Specifically, we found that a substantial portion of patients mistakenly believed that MRI involves ionizing radiation similar to X-rays and CT scans, leading to unnecessary anxiety and reluctance to undergo MRI procedures. Many patients incorrectly assumed that MRI is unsafe at any stage of pregnancy, potentially causing pregnant patients to avoid necessary diagnostic imaging. There was also a lack of awareness about what constitutes MRI-compatible and MRI-incompatible devices, which is crucial for patients with implants or devices to understand to avoid potential hazards during MRI scans. Additionally, a large percentage of patients were unaware of the need for kidney function tests before using contrast agents, leading to potential risks such as nephrogenic systemic fibrosis (NSF) in patients with impaired kidney function (23). Many patients were also unfamiliar with the four MRI zones, indicating a significant gap in understanding these essential safety measures. By addressing these gaps through targeted educational interventions and enhanced communication strategies, healthcare providers can improve patient understanding and safety during MRI procedures. This approach will help ensure patients are well-informed about MRI procedures, reducing anxiety and increasing cooperation, ultimately leading to safer and more effective MRI practices.

To address the gaps identified in this study, several strategies are recommended. Enhanced patient education initiatives, including informational brochures, videos, and workshops, should be implemented to improve patients’ understanding of MRI procedures and safety. Targeted educational interventions are needed for populations with low levels of educational attainment. In addition, healthcare providers should improve their communication strategies to ensure that patients receive clear and comprehensive information (17, 24). Training programs for radiologists, radiology technologists, and nurses should focus on enhancing interpersonal and communication skills to reduce patient anxiety. Public awareness campaigns leveraging media and community outreach can increase general knowledge about MRI and safety. Digital platforms, such as websites and mobile applications, should be utilized to disseminate accurate information to a wide audience. Implementing these recommendations can improve patient knowledge and safety regarding MRI procedures, leading to better patient outcomes and satisfaction.

The main limitation of this study was its cross-sectional design, which restricted the ability to establish causalities between patients’ sociodemographic factors and knowledge levels. In addition, the sample was drawn from only nine hospitals in various provinces of Saudi Arabia, which may limit its representativeness and the generalizability of the findings to more diverse populations. To enhance the comprehensiveness of future research, the sampling framework should be expanded to include a wider, more diverse selection of hospitals across different geographic areas. Incorporating a mixed-methods approach that combines quantitative surveys with qualitative interviews can also provide deeper insights into this research area and further validate the quantitative data.

## 5 Conclusion

The findings of this study highlight the importance of educating patients about MRI procedures and safety protocols. Although a significant proportion of participants demonstrated satisfactory knowledge, some gaps remain, particularly regarding MRI safety measures. Higher levels of educational attainment and employment status were associated with better MRI knowledge, indicating the importance of targeted educational interventions. Although healthcare professionals were the participants’ primary sources of MRI information, there is still a need for comprehensive, accessible information. Enhancing the communication strategies of healthcare providers via training programs can help improve patients’ understanding of and experiences during MRI scans.

## Data availability statement

The original contributions presented in the study are included in the article/supplementary material, further inquiries can be directed to the corresponding author.

## Ethics statement

The studies involving humans were approved by the institutional review board at King Saud University, Medical City (Reference No. E-24-8424). The studies were conducted in accordance with the local legislation and institutional requirements. The participants provided their written informed consent to participate in this study.

## Author contributions

SA: Conceptualization, Data curation, Formal analysis, Investigation, Methodology, Project administration, Resources, Software, Supervision, Validation, Visualization, Writing – original draft, Writing – review & editing.
